# Pattern of Time-to-Surgery in Patients With Breast Cancer at Different Stages of the COVID-19 Pandemic

**DOI:** 10.3389/fonc.2021.820638

**Published:** 2022-01-12

**Authors:** Ruixian Chen, Jiqiao Yang, Xin Zhao, Zhoukai Fu, Zhu Wang, Changjian Qiu, Yunhao Wu, Ruoning Yang, Weijing Liu, Ya Huang, Jie Chen

**Affiliations:** ^1^ Department of Breast Surgery, West China Hospital, Sichuan University, Chengdu, China; ^2^ Laboratory of Molecular Diagnosis of Cancer, West China Hospital, Sichuan University, Chengdu, China; ^3^ Mental Health Center, West China Hospital, Sichuan University, Chengdu, China

**Keywords:** breast cancer, time-to-surgery, COVID-19, pandemic, surgical delays, surgical oncology

## Abstract

**Background:**

The management of cancer surgeries is under unprecedented challenges during the COVID-19 pandemic, and the breast cancer patients may face a time-delay in the treatment. This retrospective study aimed to present the pattern of time-to-surgery (TTS) and analyze the features of breast cancer patients under the different stages of the COVID-19 pandemic.

**Methods:**

Patients who received surgeries for breast cancers at West China Hospital between February 15, 2020 and April 30, 2020 (the outbreak and post-peak stages), and between March 10, 2021 and May 25, 2021 (the normalization stage) were included. TTS was calculated as the time interval between the pathological diagnosis and surgical treatment of breast cancer patients. And the pandemic was divided into three stages based on the time when the patients were pathologically diagnosed and the severity of pandemic at that time point. TTS, demographic and clinicopathological features were collected from medical records.

**Results:**

A total of 367 patients were included. As for demographic features, it demonstrated statistically significant differences in insurance type (*p*<0.001) and regular screening (*p*<0.001), as well as age (*p*=0.013) and menstrual status (*p*=0.004). As for clinicopathological features, axillary involvement (*p*=0.019) was a factor that differed among three stages. The overall TTS was 23.56 ± 21.39 days. TTS for patients who were diagnosed during the outbreak of COVID-19 were longer than those diagnosed during pandemic post-peak and normalization stage (*p*<0.001). Pandemic stage (*p*<0.001) and excision biopsy before surgery (OR, 6.459; 95% CI, 2.225-18.755; *p*=0.001) were markedly correlated with the TTS of patients.

**Conclusions:**

TTS of breast cancer patients significantly varied in different stages of the COVID-19 pandemic. And breast cancer patients’ daily lives and disease treatments were affected by the pandemic in many aspects, such as health insurance access, physical screening and change of therapeutic schedules. As the time-delay may cause negative influences on patients’ disease, we should minimize the occurrence of such time-delay. It is vital to come up with comprehensive measures to deal with unexpected situations in case the pandemic occurs.

## Introduction

The COVID-19 pandemic caused by the novel coronavirus, severe acute respiratory syndrome coronavirus 2 (SARS-CoV-2), is a public health emergency of global concern, and is unprecedentedly threatening the contemporary healthcare systems (1). During the COVID-19 pandemic, managing oncological surgeries is a significant challenge. Many patients may be affected by the time delays in their treatment due to 1. Hospital running, 2. Shortage of medical supplies, 3. Traffic barriers to the patients caused by quarantine measures, and 4. Maximal social distancing (2).

Time-to-surgery (TTS) refers to the time interval between diagnosis and therapeutic surgery. Longer TTS is associated with poorer outcomes in patient with breast cancers (3, 4) and other malignancies (5, 6). In contrast, a shortened delay (approximately 30 days) is associated with survival benefits (7). Postponing specific cancer treatment may cause tumor progression, metastasis, and ultimately, cancer-related death (8). During COVID-19, the TTS among patients with breast cancer may be affected substantially. Factors such as the limited means for diagnosis and treatment, the need to prioritize the management of patients with COVID-19, and the economic impact of the COVID-19 pandemic on health system priorities and in particular, oncology (9), cumulatively increased the difficulties in managing patients with cancer.

The management of breast cancer demands special considerations during the pandemic. Although the existing studies conducted on TTS changes among patients with breast cancer during COVID-19 are mainly cross-sectional, the available evidence confirmed a time-delay in cancer treatment. High mortality risk diseases, such as cancers should not be neglected during the pandemic (10–13) and priorities should be determined. To date, there has been no studies that have dynamically analyzed the pattern of TTS changes at different stages of the COVID-19 pandemic. This study aimed to analyze the time-delay in treatment of patients with breast cancer during COVID-19, and determine the impact of the pandemic on patients by comparing patient characteristics at different stages of the pandemic. Ultimately, this study aimed to share information on the management of breast cancers during the COVID-19 pandemic.

## Materials And Methods

The study was approved by institutional review board (West China Hospital Research Ethics Committee, No. 2020[302]). Informed consent was waived due to the retrospective nature of this study.

### Patient Selection

Between February 15, 2020 and April 30, 2020, and between March 10, 2021 and May 25, 2021 (20 days after the Spring Festival, the most important traditional festival in China, in 2020 and 2021, respectively), patients with breast cancer who received surgeries at West China Hospital of Sichuan University were retrospectively identified from hospital database. The inclusion criteria were patients with breast cancers who received surgeries. The exclusion criteria were as follows: a. male patients; b. Paget’s disease; c. patients who received neoadjuvant chemotherapy; d. patients during pregnancy or lactation; e. recurrent or metastatic diseases; f. patients who have been diagnosed with other malignancies; g. patients with known major disabling medical or mental disorders.

The following information of each included patient was collected: demographic, clinical, pathological features and treatment strategies.

### Definition of Time to Surgery and the Pandemic Phases

Conventionally, TTS was calculated as the time interval between the pathological diagnosis and surgical treatment of breast cancer patients. According to TTS, patients were categorized into two groups: TTS < 40 days and TTS ≥ 40 days (3, 14).

Based on the time when the patients were pathologically diagnosed and the severity of pandemic at that time point, we categorized the patients into three groups: outbreak (diagnosed before 1.24 in 2020), post-peak (diagnosed after 1.24 in 2020) and normalization (diagnosed in 2021). As the time point of the suspension of operations, January 24, 2020 was regarded as the cut-off point for pandemic in 2020, which divided the pandemic into the outbreak and the post-peak stage. To be specific, the post-peak stage refers to a period when there was initial progress in containing the virus.

### Statistical Analysis

We used independent *t*-test and one-way analysis of variance (ANOVA) for continuous data; Pearson’s Chi-square test or Fisher’s exact test for categorical data to compare demographic, clinical and pathological features in different periods of the pandemic; binary logistic regression for univariate and multivariate analysis of TTS. A two-sided *p*-value less than 0.05 was regarded as statistically significant. All statistical analyses were performed using SPSS 21.0 (SPSS Inc., Chicago, IL, USA).

## Results

### Patterns of TTS

**Figure 1:**
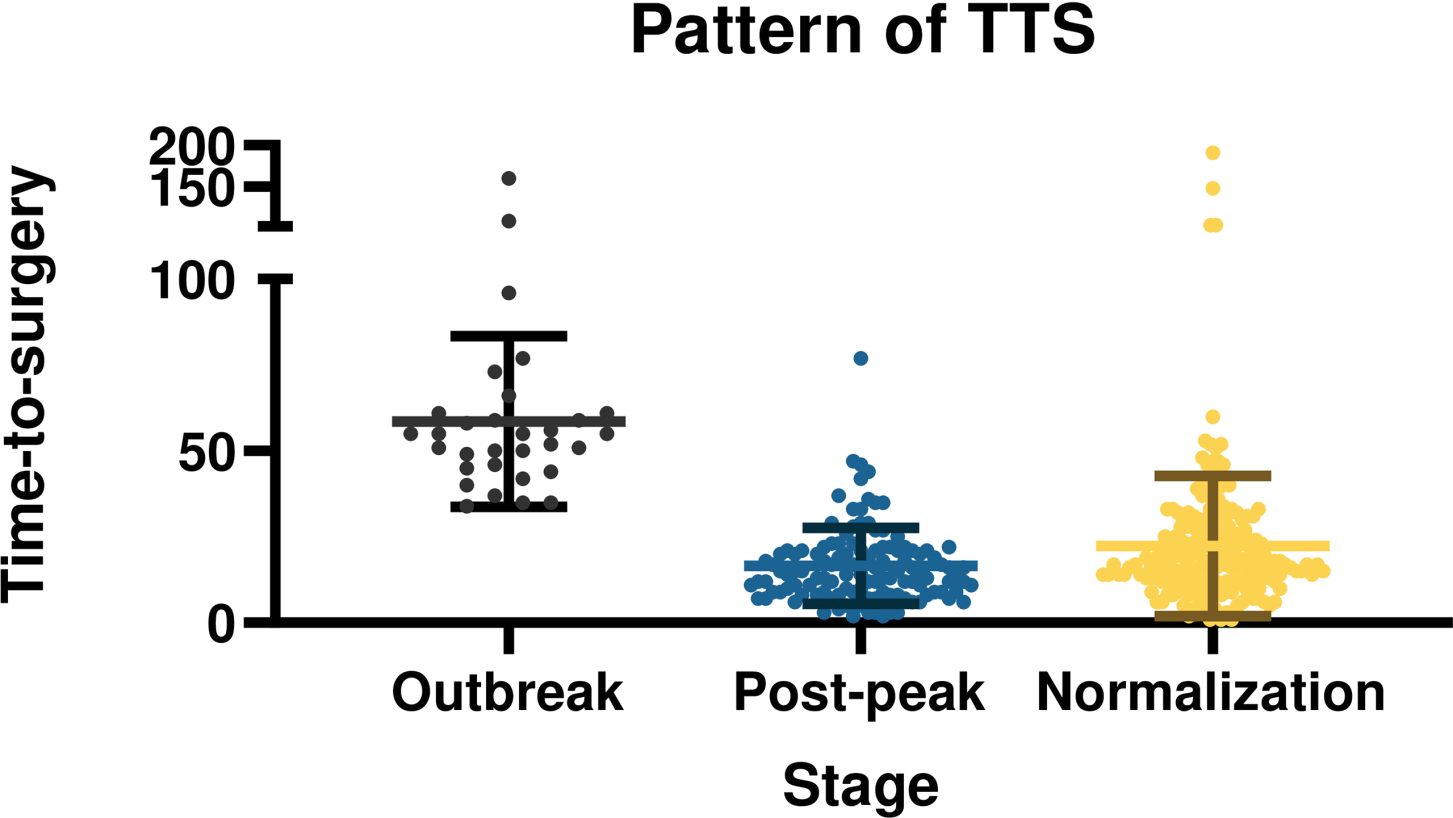
Time-to-surgery in breast cancer patients at different stages of the COVID-19 pandemic.

The overall mean TTS was 23.56 ± 21.39 days. The TTS was distinctly different at different pandemic stages (*p*<0.001). With the progression and control of the pandemic, TTS showed a pattern of first increasing, then deceasing, before finally stabilizing. The mean TTS was 58.55 ± 24.87 days during the outbreak stage of the pandemic, 16.55 ± 11.10 days in post-peak stage, and 22.38 ± 20.43 days in the normalization stage. The differences and patterns of TTS are demonstrated in **Figure 1**.

### Demographic Characteristics

In total, 367 consecutive patients met the inclusion criteria and were included for statistical analysis. None of the patient was infected with SARS-CoV-2. The mean age at diagnosis was 50.74 ± 10.63 years. The demographic features, such as setting, educational level, and marital status were not significantly different among the pandemic stages. Interestingly, statistically significant differences were found in the insurance type (*p*<0.001) and regular screening (*p*<0.001). During the normalization stage, more patients paid medical bills *via* governmental insurance and received periodic screening than patients in other stages. Patients seen in the normalization stage were generally older (*p*=0.013), and more likely to be menopausal (*p*=0.004). Detailed demographic characteristics of the patients are presented in **Table 1**.

**Table 1:** Demographic characteristics of patients by periods of pandemic.

Characteristics	Overall (n = 367)	Period according to the severity of the epidemic			*p*-value
		Outbreak (n = 31)	Post-peak (n = 129)	Normalization (n = 207)	
TTS (mean ± SD)	23.56 ± 21.39	58.55 ± 24.87	16.55 ± 11.10	22.38 ± 20.43	<0.001
Age at diagnosis (mean ± SD)	50.74 ± 10.63	46.94 ± 11.44	49.56 ± 9.70	52.04 ± 10.88	0.013
Tumor size (mm, mean ± SD)	23.11 ± 13.99	19.71 ± 11.86	24.67 ± 15.90	22.65 ± 12.90	0.161
Setting					0.113
City of the hospital	189 (51.5%)	11(35.5%)	63 (48.8%)	115 (55.6%)	
Other cities in the same province	158 (43.1%)	17 (54.8%)	56 (43.4%)	85 (41.1%)	
Other provinces	20 (5.4%)	3 (9.7%)	10 (7.8%)	7 (3.4%)	
Insurance type					<0.001
Private	27 (7.4%)	5 (16.1%)	22 (17.1%)	0 (0.0%)	
Governmental	306 (83.4%)	21 (67.7%)	99 (76.7%)	186 (89.9%)	
Uninsured	34 (9.3%)	5 (16.1%)	8 (6.2%)	21 (10.1%)	
Degree level					0.616
Junior high school or lower	159 (43.3%)	13 (41.9%)	59 (45.7%)	87 (42.0%)	
High school or college	147 (40.1%)	14 (45.2%)	45 (34.9%)	88 (42.5%)	
Bachelor degree or above	61 (16.6%)	4 (12.9%)	25 (19.4%)	32 (15.5%)	
Marital Status^a^					0.701
Married	346 (94.3%)	29 (93.5%)	120 (93.0%)	197 (95.2%)	
Unmarried^a^	21 (5.7%)	2 (6.5%)	9 (7.0%)	10 (4.8%)	
Menstrual status					0.004
Premenopausal	165 (45.0%)	22 (71.0%)	61 (47.3%)	82 (39.6%)	
Menopausal	202 (55.0%)	9 (29.0%)	68 (52.7%)	125 (60.4%)	
Regular screening					<0.001
Yes	86 (23.4%)	9 (29.0%)	14 (10.9%)	63 (30.4%)	
No	281 (76.6%)	22 (71.0%)	115 (89.1%)	144 (69.6%)	

a single/divorced/widowed/separated.

SD, standard deviation.

### Clinical and Pathological Characteristics

A significantly higher proportion of patients exhibited axillary involvement in post-peak stage than outbreak stage. On the other hand, following initial control of the virus the number of patients with axillary involvement decreased markedly during the normalization stage (*p*=0.019). Other clinicopathologic features, such as method of detection, type of surgery, AJCC stage and molecular subtype, were similar among 3 stages. Detailed clinical-pathological characteristics of the patients are presented in **Table 2**.

**Table 2:** Pathological and clinical characteristics of patients by periods of pandemic.

Characteristics	Overall (n = 367)	Period according to the severity of the epidemic			*p*-value
		Outbreak (n = 31)	Post-peak (n = 129)	Normalization (n = 207)	
Method of detection					0.131
Symptom-detected	315 (85.8%)	25 (80.6%)	117 (90.7%)	173 (83.6%)	
Screen-detected	52 (14.2%)	6(19.4%)	12 (9.3%)	34 (16.4%)	
Excision biopsy before surgery					0.652
Yes	27 (7.4%)	3 (9.7%)	11 (8.5%)	13 (6.3%)	
No	340 (92.6%)	28 (90.3%)	118 (91.5%)	194 (93.7%)	
Type of surgery					0.216
Mastectomy	300 (81.7%)	22 (71.0%)	109 (84.5%)	169 (81.6%)	
Lumpectomy	67 (18.3%)	9 (29.0%)	20 (15.5%)	38 (18.4%)	
Reconstruction after mastectomy					0.444
Yes	25 (6.8%)	3 (9.7%)	6 (4.7%)	16 (7.7%)	
No	342 (93.2%)	28 (90.3%)	123 (95.3%)	191 (92.3%)	
Histological diagnosis					0.509
Ductal	295 (80.4%)	23 (74.2%)	107 (82.9%)	165 (79.7%)	
Others/multiple	72 (19.6%)	8 (25.8%)	22 (17.1%)	42 (20.3%)	
Histological grade					0.106
1	15 (4.1%)	0 (0.0%)	5 (3.9%)	10 (4.8%)	
2	157 (42.8%)	12 (38.7%)	61 (47.3%)	84 (40.6%)	
3	140 (38.1%)	16 (51.6%)	51 (39.5%)	73 (35.3%)	
Unknown	55 (15.0%)	3 (9.7%)	12 (9.3%)	40 (19.3%)	
AJCC stage					0.094
0 & I	141 (38.4%)	14 (45.2%)	40 (31.0%)	87 (42.0%)	
II & III	226 (61.6%)	17 (54.8%)	89 (69.0%)	120 (58.0%)	
Axillary involvement					0.019
Yes	113 (30.8%)	6 (19.4%)	51 (39.5%)	56 (27.1%)	
No	254 (69.2%)	25 (80.6%)	78 (60.5%)	151 (72.9%)	
Molecular subtype					0.354
ER+/PR+, HER2-	217 (59.1%)	18 (58.1%)	67 (51.9%)	132 (63.8%)	
ER-, PR-, HER2+	40 (10.9%)	4 (12.9%)	14 (10.9%)	22 (10.6%)	
ER+/PR+, HER2+	60 (16.3%)	6 (19.4%)	27 (20.9%)	27 (13.0%)	
Triple negative	50 (13.6%)	3 (9.7%)	21 (16.3%)	26 (12.6%)	

SD, standard deviation; AJCC, American Joint Committee on Cancer; ER, estrogen receptor; PR, progesterone receptor; HER-2, human epithelial growth factor receptor 2.

### Factors That Influenced TTS

Results from the univariate analysis showed that pandemic stages (*p*<0.001), excision biopsy before surgery (OR = 4.846; 95% CI = 2.104-11.163; *p*<0.001), and menstrual status (OR = 1.836; 95% CI = 1.012-3.328; *p*=0.045) were related to the TTS (**Table 3**). Patients in the outbreak stages were more likely to have longer TTS, as were patients who received excision before surgery. No apparent associations were observed between TTS and other variables, such as age, setting, insurance type, regular screening, and molecular subtype. Results from the multivariate analysis demonstrated that the pandemic period (*p*<0.001), and excision biopsy before surgery (OR = 6.459; 95% CI = 2.225-18.755; *p*=0.001) were markedly correlated with TTS. The correlation pattern between these two factors and TTS was consistent with that in the univariate analysis.

**Table 3:** Univariate and multivariate analyses of factors influencing TTS.

Characteristics	Univariate	P value	Multivariate	P value
	OR (95% CI)		OR (95% CI)	
Period		<0.001		<0.001
Outbreak				
Post-peak	0.006 (0.002-0.026)	<0.001	0.006 (0.001-0.023)	<0.001
Normalization	0.016 (0.005-0.050)	<0.001	0.015 (0.004-0.049)	<0.001
Age	0.988 (0.960-1.016)	0.393		
Setting		0.800		
City of the hospital				
Other cities in the same province	1.189 (0.649-2.179)	0.576		
Other provinces	1.380 (0.370-5.146)	0.632		
Insurance type		0.506		
Private				
Governmental	0.834 (0.273-2.554)	0.751		
Uninsured	1.413 (0.364-5.487)	0.617		
Degree level		0.574		
Junior high school or lower				
High school or college	0.754 (0.397-1.431)	0.388		
Bachelor degree or above	0.681 (0.278-1.667)	0.400		
Marital status	0.964 (0.272-3.413)	0.955		
Regular screening	1.459 (0.799-2.662)	0.219		
Method of detection	1.533 (0.714-3.292)	0.273		
Menstrual status	1.836 (1.012-3.328)	0.045	0.919 (0.406-2.081)	0.839
Excision biopsy before surgery	4.846 (2.104-11.163)	<0.001	6.459 (2.225-18.755)	0.001
Type of surgery	1.752 (0.887-3.460)	0.106		
Reconstruction after mastectomy	1.802 (0.635-5.118)	0.269		
Histological diagnosis	0.624 (0.317-1.228)	0.172		
Histological grade		0.960		
1				
2	0.752 (0.144-3.926)	0.735		
3	0.803 (0.344-1.872)	0.611		
Unknown	0.815 (0.343-1.937)	0.643		
AJCC stage	0.814 (0.448-1.479)	0.499		
Axillary involvement	0.567 (0.279-1.150)	0.116		
Molecular subtype		0.915		
ER+/PR+, HER2-				
ER-, PR-, HER2+	1.232 (0.501-3.030)	0.650		
ER+/PR+, HER2+	0.813 (0.338-1.956)	0.644		
Triple negative	0.968 (0.399-2.348)	0.942		

AJCC, American Joint Committee on Cancer; ER, estrogen receptor; PR, progesterone receptor; HER-2, human epithelial growth factor receptor 2.

## Discussion

The COVID-19 pandemic dramatically impacts people globally. The first case of COVID-19 in the Sichuan province of China was confirmed on January 21, 2020. Surgeries at the Department of Breast Surgery, West China Hospital, the largest hospital in western China, have been temporarily suspended since January 24, 2020 (the national Chinese Spring Festival holiday). Starting from mid-February, surgeries were gradually resumed on the basis of limited admissions. By April 2020, the daily running of the hospital (diagnosis and treatment) had generally returned to normal.

The TTS of patients with breast cancer was the longest during the COVID-19 outbreak stage and the shortest at the post-peak stage. In contrast, the TTS during the normalization stage in 2021 was moderate. In the beginning of the pandemic, transportation between cities and provinces were temporarily cut off to stop the virus from spreading, which led to expected diagnostic and treatment delays. Moreover, little was known about the virus at the start of the pandemic, and the fear of COVID-19 led many to voluntarily and completely avoid social contact, which may be the main reason for the increased TTS.

Surprisingly, the TTS dropped shortly after the outbreak stage of the pandemic, to a duration shorter than that in the normalization stage. This may be due to patients choosing to attend local hospitals instead of waiting to be admitted in large hospitals. Further, due to the limited access to surgeries at the time, selected patients with operable breast cancers received neoadjuvant chemotherapy instead of surgeries. Consequently, the number of patients waiting for treatment in the hospital reduced, and the patients might be seen and treated more quickly during post-peak stage. The TTS of the normalization stage in 2021 is likely to be maintained for a long time into the future. Many previous studies have reported on the delay associated with cancer surgeries during the COVID-19 pandemic. The time-delay ranged from 1−2 weeks to several months. The risk of patient exposure, turnover times, the patient’s fear, and economic instability could all influence the TTS (8, 9, 15). This study was conducted to provide more in-depth analysis of the differences in TTS at different stages of COVID-19.

Age was a key patient characteristic that was significantly different among the different stages of the pandemic. Patients in the normalization stage were on average, older. Concerning the aging process, older patients with breast cancer tend to have more comorbidities and worse physical conditions than the younger patients (16). It is possible that the older patients chose to avoid infection risks by not going to the hospital during the pandemic. The pandemic led to many inconveniences for patients with breast cancer, including health insurance access and physical screening. Some public departments including government health insurance institutions, had limited service-delivery during the outbreak, therefore many patients experienced difficulties in accessing governmental insurance to pay for their hospital fees. In addition, patients could not access regular screening when the pandemic led to a suspension of routine examinations in many of the medical institutions. It is also worth noting that the axillary involvement of patients differed throughout the three pandemic stages. The difference is different from expected, which may be due to the deviation caused by the small sample size of the outbreak stage. This conclusion needs to be verified by the results of larger samples.

Most of the potential influencing factors of TTS turned out to be irrelevant variables. Meanwhile, different stages of the pandemic accounted for most of the differences in the TTS. During the different stages of the pandemic, not only did the hospital and surgeries run differently, but the patients also experienced different events and possessed different mindsets. In the initial stage of the pandemic, the suspension of hospital operations, diminished healthcare resources, and patient’s resistance to social contact all contributed to the longer TTS. Garcia, et al. also found that delays in oncologic surgery negatively affected many tumor types (11). While the impact of delays on the outcomes in each cancer subtype was complex, cancers that were primarily treated surgically were the most compromised due to treatment delays. As reported, each 30-day increase in TTS elevated the risk of death by 10% for patients with breast cancer (7). Another study highlighted that oncologic surgery should be scheduled in a timely fashion due to the tendency for tumor volume to increase within 1–3 months (17). Delays in tumor management may result in disease progression. As such, hospitals need to respond to the challenges of the pandemic rapidly by reducing TTS. To avoid the negative impact of longer TTS on the prognosis of patients with breast cancer, several studies have recommended that patients with invasive breast cancer should be classified with multidisciplinary measures and assessed for the potential risks of receiving neoadjuvant therapies during the pandemic (18, 19). Neoadjuvant chemotherapies could also be a temporary alternative to surgical treatment. Another factor which influenced TTS was whether patients had an excision biopsy. Excision biopsy before surgery led to longer TTS. Patients who underwent an excision biopsy before surgery are generally expected to receive further treatment and advice from a tertiary hospital. However, transfer from local hospitals and consultations at large hospitals involve time delays. Hence, these patients tended to have longer TTS. To reduce TTS, the continuation of treatment at local hospitals may also be an option.

Some experts have suggested that it is appropriate to delay surgery during COVID-19. The European Society for Medical Oncology (ESMO) recommended that the initial operation of low-risk early breast cancer could be postponed for up to 12 weeks (20). Tseng, et al. also advised postponing surgery to optimize manpower and resource reallocation (21). Nonetheless, the negative impact of delay in TTS should be considered. In Italy, a multidisciplinary approach for breast cancer care successfully reduced both COVID-19 infections and time-delay of surgeries (22). They implemented measures such as deferring scheduled surgeries of benign disease, patients staying in single room, no accompanying relatives in the ward, and necessary protective procedures.

During the COVID-19 pandemic outbreak, a lack of adequate advanced preparation had led to time-delays in cancer treatment. Breast cancer centers should ensure thorough protective measures, adequate social distancing, and quali-quantitative standard of breast cancer care to be well-prepared for potential and anticipated challenges. Our study provided a perspective of how the COVID-19 pandemic affected patients with breast cancer by prolonging the TTS, which in turn inspired us to develop comprehensive methods for challenges such as a pandemic.

There are several limitations to this study. Firstly, the TTS can be largely affected by the severity of local epidemics and local government policies. Thus, our findings must be interpreted with the local conditions in mind. Secondly, the retrospective design of this study may inevitably bias patient selection. Thirdly, this is a single-center study, and the sample size was relatively small.

In conclusion, the current study presented the pattern of TTS for patients with breast cancers during the COVID-19 pandemic and shared the experience of a single center. Future studies with larger sample populations are warranted to validate our results.

## Data Availability Statement

The raw data supporting the conclusions of this article will be made available by the authors, without undue reservation.

## Author Contributions

JC, RC, and JY contributed to conception and design of the study. RC and JY performed the statistical analysis. RC and JY wrote the first draft of the manuscript. All authors contributed to manuscript revision, read, and approved the submitted version

## Funding

This study is supported by the funding from the National Natural Science Foundation of China (32071284) and the Fundamental Research Funds for the Central Universities (2021SCU12021).

## Conflict of Interest

The authors declare that the research was conducted in the absence of any commercial or financial relationships that could be construed as a potential conflict of interest.

## Publisher’s Note

All claims expressed in this article are solely those of the authors and do not necessarily represent those of their affiliated organizations, or those of the publisher, the editors and the reviewers. Any product that may be evaluated in this article, or claim that may be made by its manufacturer, is not guaranteed or endorsed by the publisher.
